# Fatigue in patients with hereditary neuropathy with liability to pressure palsies

**DOI:** 10.1002/acn3.51133

**Published:** 2020-07-28

**Authors:** Nora E. Fritz, Yongsheng Chen, Lauren Waters, Sadaf Saba, Melody Hackett, Flicia C. Mada, Jun Li

**Affiliations:** ^1^ Physical Therapy Program Eugene Applebaum College of Pharmacy and Health Sciences Detroit MI; ^2^ Department of Neurology Wayne State University School of Medicine Detroit MI; ^3^ Center for Molecular Medicine & Genetics Wayne State University School of Medicine Detroit MI; ^4^ John D. Dingell VA Medical Center Detroit MI

## Abstract

**Objective:**

Hereditary Neuropathy with Liability to Pressure Palsies (HNPP) is caused by a heterozygous deletion of peripheral myelin protein‐22 (*PMP22*) gene resulting in focal sensorimotor deficits. Our lab has identified a disruption of myelin junctions in excessively permeable myelin that impairs action potential propagation. This mechanism is expected to cause fatigue in patients with HNPP. Therefore, the objective was to characterize fatigue in patients with HNPP and determine the relationship of fatigue to nerve pathology, disability, and quality of life.

**Methods:**

Nine females with HNPP participated in a single visit that included genotyping, nerve conduction studies, neurological exam, quantitative magnetic resonance imaging, and a physical therapy exam incorporating upper and lower extremity function and survey measures of fatigue. This visit was followed by 2 weeks of ecological momentary assessment (wrist‐worn device) that captured fatigue ratings five times per day.

**Results:**

Participants demonstrated mild neurological impairment (CMTNS: 5.7 ± 2.8), yet reported high fatigue levels (average fatigue intensity over 2 weeks: 5.9 out of 10). Higher fatigue levels were associated with poorer quality of life and more pain. Higher fatigue was associated with significantly greater distal nerve proton density changes on peripheral nerve MRI, which is in line with hyper‐permeable myelin in HNPP.

**Interpretation:**

Fatigue is common and severe among patients with HNPP whose disabilities are minimal by conventional outcome measures. Therapeutic interventions targeting fatigue have the potential to improve quality of life and may serve as a robust outcome measure to show longitudinal changes for patients with HNPP.

## Introduction

Hereditary neuropathy with liability to pressure palsies (HNPP) is caused by heterozygous deletion of *peripheral myelin protein‐22* (*PMP22*) gene.^1^, ^2^, ^3^ Patients typically present with focal sensory motor deficits. These episodes are often brought on by mild physical activities that are innocuous to healthy people. Symmetric sensory motor polyneuropathy may develop in elderly with HNPP.^4^ Asymptomatic patients may develop severe limb paralysis after strenuous physical activities such as running long distances with heavy loads.^5^ This imposes a catastrophic risk in a fraction of patients with HNPP.

Our laboratory has demonstrated an abnormally increased permeability of myelin in an HNPP mouse model (*Pmp22^+/‐^*) due to the disruption of junctions that seal the spaces between laminae of myelin. This impairs action potential propagation in the absence of demyelination (stripping myelin off the axon), called functional demyelination,^6^, ^7^ which may cause muscle fatigue and/or weakness.

Pathological fatigue, marked by exhaustion that is more than just tiredness, has been described in persons with other demyelinating diseases, such as multiple sclerosis,^8^ which contributes to increasing disability. Although persons with HNPP commonly report fatigue, it has not been systematically studied. Functional demyelination in HNPP leads us to hypothesize that the fatigue is likely a debilitating feature in patients with HNPP. Moreover, we have identified a small molecular compound to improve the functional demyelination in our preclinical studies.^6^ This discovery further motivates us to examine fatigue in HNPP. Results from this study may be informative for designing future therapeutic trials targeting HNPP fatigue.

## Methods

### Subjects

Participants (9 female patients, age ranging from 30 to 67 years) were recruited through the Wayne State University Charcot‐Marie‐Tooth (CMT) Clinic where the author (JL) serves as the Director. All testing occurred in a single visit. All participants signed an informed consent prior to testing, and all testing procedures were approved by the Wayne State University Institutional Review Board.

### Genotyping

HNPP was diagnosed in all participants by the genetic test (multiplex PRC) showing a heterozygous deletion of *PMP22*.

### Nerve conduction studies (NCS)

NCS were done as previously described.^9^ In brief, for motor nerves in the arms and legs, distal stimulation was done at 7 cm and 9 cm, respectively. The stimulation distance for the sensory nerves in median, ulnar, and sural nerves was 14 cm, but for the radial nerve it was 11 cm.

### CMT neuropathy score (CMTNS)

Medical history and neurological exam were collected from each patient. CMTNS is comprised of sensorimotor symptoms, physical findings in limbs as well as nerve conduction studies.^10^ Therefore, CMTNS was obtained from all patients as a measure of clinical impairment.

### Quantitative Magnetic Resonance Imaging (qMRI)

In addition to the participants with HNPP, we recruited four additional female healthy controls (HC, age ranging from 20 to 65 years) for qMRI study. These HCs have no known history of neurological disease. All subjects were scanned on the same 3T system (Verio, Siemens Healthineers, Erlangen, Germany) with an eight‐channel knee coil positioned at two boney marker locations: mid‐thigh level proximal to the knee, and mid‐calf level distal to the knee. An established imaging protocol was performed in the axial plane.^11^ It includes: ***i)*** an interleaved two‐point Dixon three‐dimensional (3D) gradient recalled echo (GRE) scan for muscle fat fraction (FF);^12^, ^13^ ***ii)*** a high‐resolution 3D GRE scan for nerve fascicular cross‐section area (fCSA); ***iii)*** the strategically acquired gradient echo (STAGE) imaging^14^ for nerve longitudinal relaxation time (T1) and proton density (PD) mappings, which also generates the transmitter and receiver radiofrequency field maps for correcting heterogeneities in the T1, PD and magnetization transfer ratio (MTR) maps; and ***iv)*** two 3D GRE scans performed with and without MT pulses for nerve MTR and effective transverse relaxation time (T2star).

MRI data were processed using an in‐house developed program in MATLAB (R2019a, MathWorks, Natick, MA, USA) with image alignments via SPM (fil.ion.ucl.ac.uk/spm/).

This established processing pipeline^11^ generates six qMRI metrics, including the mean of FF for muscle, as well as means of fCSA, T1, PD, MTR, and T2star for peripheral nerves (sciatic and tibial for this study). Previously described semi‐automated segmentation methods^11^ were used for extracting volumetric muscle FF and the other 5 indices for nerves at the central slice. The nerve PD was normalized by the adjacent normal‐appearing muscle, because the PD map depends on the reconstruction scale which is different for each MRI scan.

### Measures of Function

Participants completed the 10‐meter walk test for gait speed assessment, 9‐hole peg test for upper extremity dexterity, as well as a battery of survey measures to examine quality of life (SF‐36), pain (PROMIS pain scale), depression (NeuroQoL‐Depression scale) and sleep (NeuroQol‐Sleep scale). Neuro‐QoL and PROMIS measures were developed and validated to evaluate physical, mental, and social effects experienced by adults living with chronic neurological conditions.^15^

### Measures of fatigue

We defined pathologic fatigue^16^ as exhaustion or severely impaired endurance in performing daily activities that would not lead to exhaustion or abnormal endurance in a normal person. Typically, fatigue is assessed with self‐report scales, though our recent work^17^ supports the use of Ecological Momentary Assessment (EMA), in which fatigue is reported multiple times per day over weeks in the individual’s natural environment. EMA provides a better view of the dynamic nature of fatigue and limits recall bias. Thus, we assessed fatigue with both a self‐report scale and EMA.

The NeuroQoL Fatigue Short Form^15^ is an eight‐item self‐report survey with a possible 40 points, where higher scores indicate worse fatigue.^18^ The NeuroQoL‐Fatigue has established validity in neurologic populations and is recommended as a part of the NIH toolbox.

The NeuroQoL Fatigue was supplemented by Ecological Momentary Assessment (EMA), where participants wore a wrist‐worn device, the ProDiary (CamNTech, Cambridge, UK; about the size of a watch) that prompted the user to report fatigue levels five times per day (wake‐up, mid‐morning, mid‐afternoon, evening, bedtime) over a 2‐week period following the in‐person visit. Participants were asked to rate both the intensity of fatigue (“what is your level of fatigue?”) and impact of fatigue (“how much is fatigue interfering with your daily activity right now?”) on a 0‐10 numerical rating scale entered directly on the ProDiary. EMA measures have been shown to provide a more reliable and sensitive assay of symptoms compared to traditional recall reports^19^ as they capture fluctuations and variability in symptoms over time. Participants mailed the ProDiary back in a pre‐paid USPS box. Average scores for fatigue ratings over the 2‐week period were calculated. There were no cases of severe limb paralysis during the 2‐week monitoring period, and fatigue scores are unlikely affected by transient focal episodes.

### Measure of activity

The ProDiary device was outfitted with accelerometry for examination of activity levels and mobility throughout the 2‐week period. Average scores for activity were calculated.

### Statistics

SPSS version 26 was used for fatigue data analysis. Spearman correlations were used to determine the relationship among fatigue measures and measures of function. Given the small sample size of persons with HNPP, we evaluated r‐values (rather than p‐values) for potential impact of relationships between variables. MATLAB was used for statistical analyses on qMRI data and the correlations between qMRI indices and fatigue measurements: (1) Wilcoxon rank‐sum test with a threshold of *P < 0.05* as significant, to evaluate differences between HC and HNPP for each qMRI index; (2) linear regression model by fitting to data considering strong correlation effect for *r^2^>=0.7*, moderate effect for *0.7> r^2^>=0.5*, and weak (or no) effect for *r^2^ < 0.5*, each with a threshold of *P < 0.05* as significant model.

### Data Availability

Anonymized data will be shared by request from any qualified investigator.

## Results

### Genotype and phenotype in the HNPP cohort

Nine women participated the study (average age: 50.5 ± 11.0 SD; years of education: 16.1 ± 3.7). None used any assistant devices, such as ankle or wrist brace. All participants had a genetically confirmed heterozygous deletion of *PMP22*.

All patients with HNPP reported 1–3 incidents of reversible focal sensory loss and weakness throughout their life, which may or may not be triggered by mechanical compression. Participants reported that these episodes took from weeks to more than a year to recover.

NCS showed focally slowed nerve conduction at the sites susceptible to mechanical pressure. For instance, six patients had a prolonged distal latency of the median motor nerve across the wrist (average of all patients: 4.91 ± 0.98 ms in Table 1), but only 1 of the nine had a prolonged distal latency in ulnar motor nerve across the wrist (average of all patients: 3.34 ± 0.64 ms in Table 1. In addition, a focal slowing of conduction velocities was observed in the ulnar motor nerves across the elbow in six patients and in the peroneal motor nerve across the fibular head in all patients. These features suggest this cohort manifesting a typical HNPP phenotype.

**Table 1:** Electrophysiological findings of the studied participants

Code #	Sensory Nerve Conduction, DL/Amp/CV^a^				Motor Nerve Conduction, DL/Amp/CV			
	Median	Ulnar	Radial	Sural	Median	Ulnar	Peroneal	Tibial
Norm^b^	3.5/22/50	3.5/10/50	2.7/10/48	4.4/6.0/40	4.4/4.0/49	3.3/6.0/49	6.5/2.0/44	2.0/6.1/40
0025	3.85/18.1/48.9	4.22/14.4/43.4	2.34/21.2/60.0	4.43/5.0/39.5	3.91/9.2/53.2	3.07/11.2/54.4	6.82/1.5/39.9	4.64/5.3/39.7
0026	6.30/9.7/28.6	4.27/12.2/41.4	2.71/16.1/51.9	4.53/3.1/37.9	5.99/5.4/42.9	3.18/9.2/55.1	8.39/1.9/37.1	4.79/2.9/40.6
0027	5.36/13.4/34.9	4.11/11.3/44.8	2.97/18.5/45.7	NA^c^	5.36/7.6/47.4	2.86/9.5/54.0	5.83/3.0/40.8	5.73/2.2/44.8
0028	4.22/17.5/41.4	3.75/9.6/48.9	2.66/23.8/50.5	NA	4.58/7.7/53.6	2.97/10.8/53.6	5.31/2.3/39.3	5.99/6.9/41.7
0029	4.84/8.3/39.5	5.63/11.3/33.2	3.91/8.5/33.7	5.36/2.2/33.6	5.31/8.5/27.5	5.10/5.9/50.1	9.11/3.1/38.9	5.16/4.0/41.0
0030	6.2/11/27.7	NR^d^	2.66/11.2/53.3	NR	6.77/11.5/50.3	3.33/10/54	5.31/8.3/40.8	5.68/9/49.9
0031	4.53/26.4/40.1	4.11/29.5/44.8	2.60/24.2/58.2	4.79/10.3/38.4	4.58/7/50.2	3.28/12.5/52.1	5.42/4.1/38.9	5.68/10.2/47.1
0032	3.54/22.7/54.9	3.85/18.5/50.7	2.55/15.4/54.9	4.38/4.4/41.4	3.65/7/54.5	3.28/10.1/52.9	5/4.2/40.9	5.47/5.3/43.7
0033	4.27/14.2/43.4	3.80/13.6/48.0	2.60/22.9/58.2	3.91/5.3/48.0	4.01/5.6/54.5	2.97/9.8/55.3	5.16/3.2/43.1	4.17/14.8/42.9

^a^ DL/Amp/CV, Distal Latency/Amplitude/Conduction Velocity,

^b^ Norm, Normative values,

^c^ NA, Not Available.

^d^ NR, Not Responsive.

All participants were evaluated by the CMTNS with a mean of 5.67 ± 2.75. Previous studies^20^ have classified the CMT severity into three categories based on CMTNS (mild (CMTNS ≤ 10), moderate (11 to 20), and severe (≥21)). Thus, all participants had a mild phenotype. In particular, we observed no or minimal muscle weakness on physical examination. For instance, all patients had 5 on the Medical Research Council (MRC) scale in all muscles, including ankle dorsal flexors and hand intrinsic muscles. Therefore, this cohort was ideal to test fatigue since weakness will not be confused with fatigue.

### Fatigue and its correlation with other functions in patients with HNPP

Eight of nine participants completed fatigue assessments. Subjective report of fatigue at a single time point (NeuroQol‐Fatigue) was moderately correlated with average weekly subjective reports of fatigue [EMA measures of fatigue intensity (*r* = 0.500) and interference (*r* = 0.500)]; however, weekly subjective fatigue intensity and interference were strongly related (*r* = 0.929; *P* = 0.001) (Table 2). Fatigue severity and fatigue intensity scores ranged from 0 to 10. This suggests that fatigue in persons with HNPP may vary over the course of a week, which could skew the data if collected at a single time point unless the sample size is large enough.

**Table 2:** Functional, Fatigue and Activity Measures in n = 8 participants with HNPP.

Measure	Domain	Mean (SD)
*Survey Measures*		
SF‐36	Quality of Life	38.8 ± 16.0 0.91 ± 0.27 0.42 ± 0.43 20.6 ± 15.5 71.0 ± 20.3 54.7 ± 18.8 39.4 ± 10.8 34.4 ± 18.8
	Physical Functioning	
	Role Limitations – Physical Health	
	Role Limitations – Emotional Health	
	Energy/Fatigue	
	Emotional Well‐Being	
	Social Functioning	
	Pain	
	General Health	
PROMIS Pain	Pain	8.4 ± 3.9
NeuroQol‐Depression	Depression	11.4 ± 3.7
Neuro‐Qol‐ Sleep	Sleep	24.6 ± 6.9
NeuroQol‐ Fatigue	Fatigue	33.6 ± 5.6
*EMA Measures*		
Patient Report	Fatigue Intensity	5.9 ± 1.9
Patient Report	Fatigue Interference	5.3 ± 2.5

EMA weekly average fatigue intensity and interference scores were strongly related to the general health quality of life subscale of SF‐36 (*r* = −0.955 and *r* = −0.919, respectively) suggesting that greater fatigue is associated with poorer quality of life. Greater fatigue intensity was also related to poorer ratings in other domains of quality of life, including more role limitations in physical health (*r* = 0.486), more role limitations in emotional health (*r* = 0.433), poorer overall energy quality of life (*r* = −0.618); poorer emotional well‐being (*r* = −0.733); poorer social functioning (*r* = −0.537) and worse pain (*r *= −0.604).

Finally, a moderate relationship was seen between greater fatigue intensity and higher pain ratings (*r* = 0.509) on the PROMIS Pain scale. No relationship was noted between fatigue levels and depression, sleep, gait, activity levels, or hand functioning in this cohort.

### Fatigue in patients with HNPP was not correlated with axonal loss but with distal nerve proton density changes on MRI

Our previous work has demonstrated a mild axonal loss in patients with HNPP using qMRI.^21^ The axonal loss was quantified by the muscle FF. After axon degenerates, fat increases in denervated muscle.^21^ We therefore tested whether the axonal loss is related to the fatigue.

**Figure 1:**
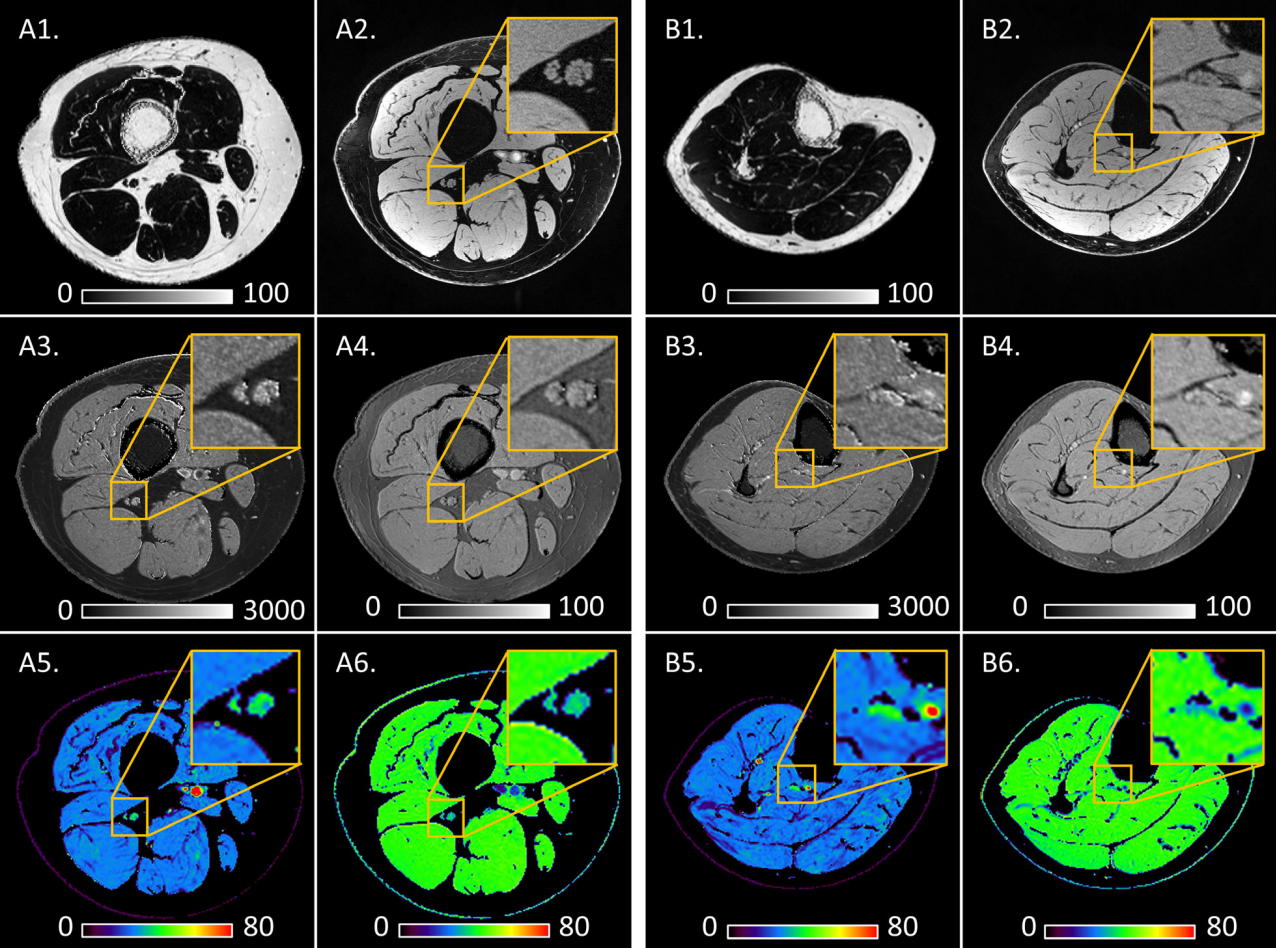
Representative MRI images. These representative data were obtained from a patient with HNPP (52‐year‐old women) at mid‐thigh (panel A) and mid‐calf (panel B) levels. A1 (B1) is thigh (calf) muscle FF image. A2 (B2) is high‐resolution anatomical images for measuring sciatic (tibial) nerve fascicular cross‐sectional area (fCSA). A3 to A6 (B3 to B6) are quantitative images for measuring sciatic (tibial) nerve T1, PD, T2* and MTR, respectively. Sub images on each one were 3x‐zoomed local images on the nerve territories. Images were acquired with 3 mm slice thickness and in‐plane resolution 0.15 x 0.15 mm^2^ for fCSA; 0.3 x 0.3 mm^2^ for T1 and PD; and 0.6 x 0.6 mm^2^ for FF, T2* and MTR. (Units – FF: %; fCSA: mm^2^; T1: ms; PD: a.u.; T2*: ms; MTR: %).

Eight of nine HNPP participants completed MRI assessments. qMRI was performed at mid‐thigh and mid‐calf to image both muscles and nerves. Representative MRI images are shown in Figure 1. Two of the eight HNPP participants were scanned only at mid‐calf due to body size. Demographic and MRI data are shown in Table 3.

**Table 3:** Quantitative MRI data.

	HC	HNPP	*P*‐value*
Thigh session:	(*n* = 4)	(*n* = 6)	
Age (years)	43.8 ± 20.2 (20.0–65.0)	52.7 ± 5.8 (42.0–58.0)	0.648
Female percentage	100%	100%	‐
Thigh Muscle ‐ volFF (%)	7.0 ± 0.7 (6.2–7.6)	7.8 ± 1.5 (6.5–10.1)	0.610
Sciatic Nerve – fCSA (mm^2^)	17.0 ± 1.0 (15.9–18.0)	17.4 ± 3.0 (13.3–22.2)	1.000
Sciatic Nerve ‐ T1 (ms)	1497.1 ± 46.0 (1447.0–1557.6)	1542.2 ± 214.2 (1233.7–1742.8)	0.476
Sciatic Nerve – PD (a.u.)	96.8 ± 1.1 (95.9–98.4)	101.4 ± 9.7 (90.3–111.9)	1.000
Sciatic Nerve ‐ T2star (ms)	31.6 ± 4.3 (27.9–37.5)	32.4 ± 3.8 (27.7–36.2)	1.000
Sciatic Nerve ‐ MTR (%)	38.1 ± 2.0 (35.6–39.8)	38.9 ± 2.9 (34.3–41.7)	0.476
Calf session:	(n = 4)	(n = 8)	
Age (years)	43.8 ± 20.2 (20.0–65.0)	51.6 ± 11.2 (30.0–67.0)	0.610
Female percentage	100%	100%	‐
Calf Muscle ‐ volFF (%)	6.3 ± 1.0 (5.0–7.2)	8.6 ± 2.0 (6.5–13.2)	0.016*
Tibial Nerve – fCSA (mm^2^)	6.8 ± 1.0 (5.8–7.7)	6.6 ± 1.1 (5.5–8.4)	0.570
Tibial Nerve ‐ T1 (ms)	1524.0 ± 118.5 (1387.4–1661.2)	1604.0 ± 154.0 (1394.4–1901.4)	0.461
Tibial Nerve – PD (a.u.)	89.2 ± 2.2 (86.4–91.7)	97.3 ± 5.2 (89.5–103.8)	0.008*
Tibial Nerve ‐ T2star (ms)	32.0 ± 2.1 (29.9–34.4)	32.8 ± 5.1 (25.1–39.5)	0.683
Tibial Nerve ‐ MTR (%)	37.3 ± 2.3 (33.9–39.1)	38.1 ± 3.1 (33.1–42.3)	0.570

HC, Healthy control; HNPP, Hereditary neuropathy with liability to pressure palsies; volFF, Muscle volumetric fat fraction; fCSA, Fascicular cross‐sectional area; T1, Longitudinal relaxation time; PD, Proton density (PD ratio between nerve and adjacent normal‐appearing muscle); T2star, Effective transverse relaxation time; MTR, Magnetization transfer ratio.

* = statistically significant.

**Figure 2:**
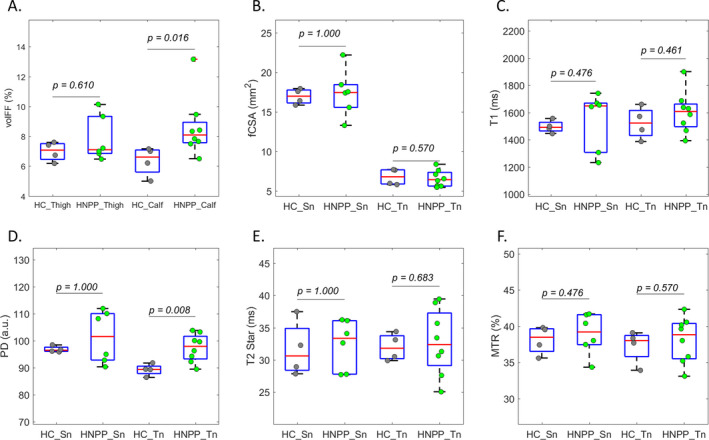
qMRI results. We recruited eight participants (all female, 30–67 years old) with HNPP and four healthy control (HC, all female, 20–65 years old). Two participants with HNPP were not scanned at thigh level due to patient size. Wilcoxon rank‐sum tests were performed to compared the two cohorts for volumetric muscle fat fraction (volFF, A) on thigh and calf muscles; as well as fascicular cross‐sectional area (fCSA, B), T1 (C), proton density (PD, D), T2* (E) and magnetization transfer ratio (MTR, F) for sciatic nerve (Sn) and tibial nerve (Tn), respectively. Compared with HC: volFF was significantly elevated in HNPP on distal muscle but not proximal muscle; the PD was also significantly elevated in HNPP on Tn but not Sn. There were no significant differences for other tests between the two cohorts.

In agreement with our previous results^20^, muscle FF in patients with HNPP was significantly elevated (*P = 0.016*) at calf level, but not thigh level (*P = 0.61*), compared with controls, confirming an axonal loss in a length dependent fashion. In line with muscle FF finding, distal (tibial) nerve proton density (PD), but not proximal (sciatic) nerve PD, was significantly elevated (*P = 0.008*), compared with controls (Figure 2 and Table 3). Values in MTR, T1, and R2* were not significantly different between patients and controls. This was in line with very low values of CMTNS and mildly increased muscle FF in all patients.

**Figure 3:**
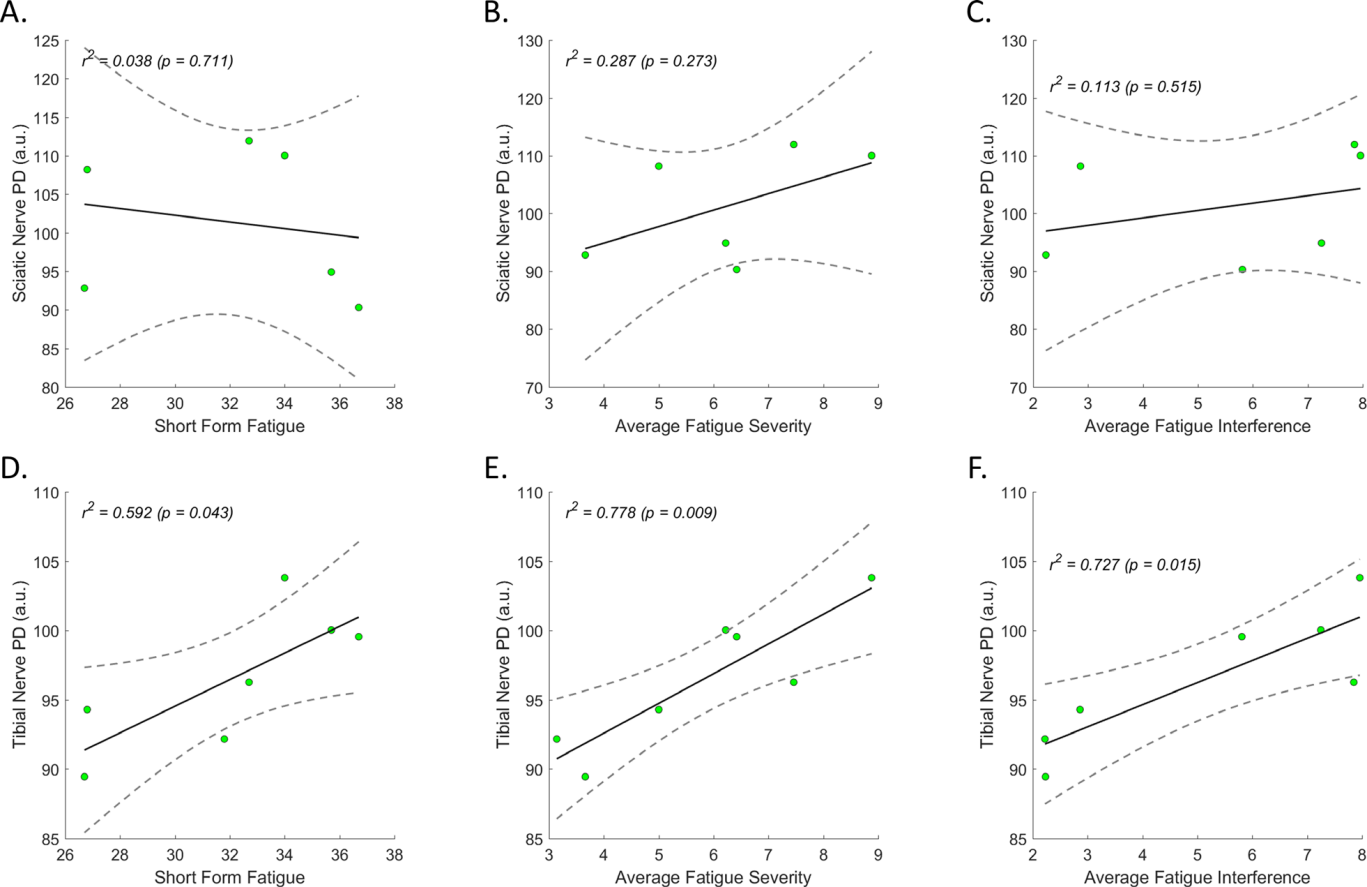
Distal nerve proton density correlated with fatigue. Linear regression model correlation analyses were performed for fatigue data versus all qMRI indices in participants with HNPP, respectively. There were strong and positive correlations between tibial nerve proton density (PD) versus average fatigue severity (E) and interference (F), and moderate and positive correlation between short form fatigue score and tibial nerve PD (D), but no effects on tests for proximal (sciatic) nerve proton density (A to C). There were also no effects for tests between fatigue measurements versus other qMRI indices including muscle FF, nerve fascicular cross‐sectional area, T1, T2* and magnetization transfer ratio (MTR).

These qMRI indices were then correlated with the fatigue scores statistically. There were no significant correlations found between muscle FF and fatigue scores. Instead, there were strong and positive correlations for distal (tibial) nerve PD versus average fatigue severity (*r^2^ = 0.778, P = 0.009*) and interference (*r^2^ = 0.727, P = 0.015*) measured with EMA (Figure 3). The distal (tibial) nerve PD also correlated with short form fatigue score (*r^2^ = 0.592, P = 0.043*) with a moderate and positive effect (Figure 3). There were also no correlations between fatigue scores and other qMRI indices including nerve fCSA, T1, T2star and MTR.

## Discussion

Our study has observed that fatigue greatly impacts persons with HNPP. On average, patients with HNPP reported 33.6 ± 5.6 on the NeuroQol‐Fatigue short form out of a maximum of 40 points. The average fatigue rating over a week was 5.9 out of 10 and interference rating was 5.3 out of 10. By comparison, our recent work in persons with multiple sclerosis with significant fatigue, average weekly fatigue ratings were between 3.9 and 4.4 at baseline, while average fatigue interference ratings were between 3.0 and 3.1 at baseline.^17^

This was an unexpected result. Patients with multiple sclerosis may develop significant neurological disabilities due to lesions in the central nervous system. For instance, in a recent study that enrolled persons with multiple sclerosis with significant fatigue, the multiple sclerosis disability score (PDDS) was at a median of 3.0 (range 1–5) in these patients,^22^ suggesting moderate disabilities. Yet, their fatigue scores were much than patients with HNPP, who showed minimal disabilities by CMTNS values. The low values of CMTNS are also in line with the mild axonal loss by the FF test and normal measurements in most nerve qMRI indices.

Among all those qMRI indices, PD appears to be the most sensitive measure to detect nerve pathology in patients with HNPP. It is known that the MRI PD is linearly correlated to the tissue water content. We speculate the increased permeability of myelin in HNPP causes increased water content between myelin lamina, but overall myelin density remains unchanged. This is consistent with the MTR data reflecting myelin density that was not significantly different in HNPP compared to controls. Therefore, such conspicuous discrepancy between subtle neurological disabilities and severe fatigue demands explanation in patients with HNPP.

**Figure 4:**
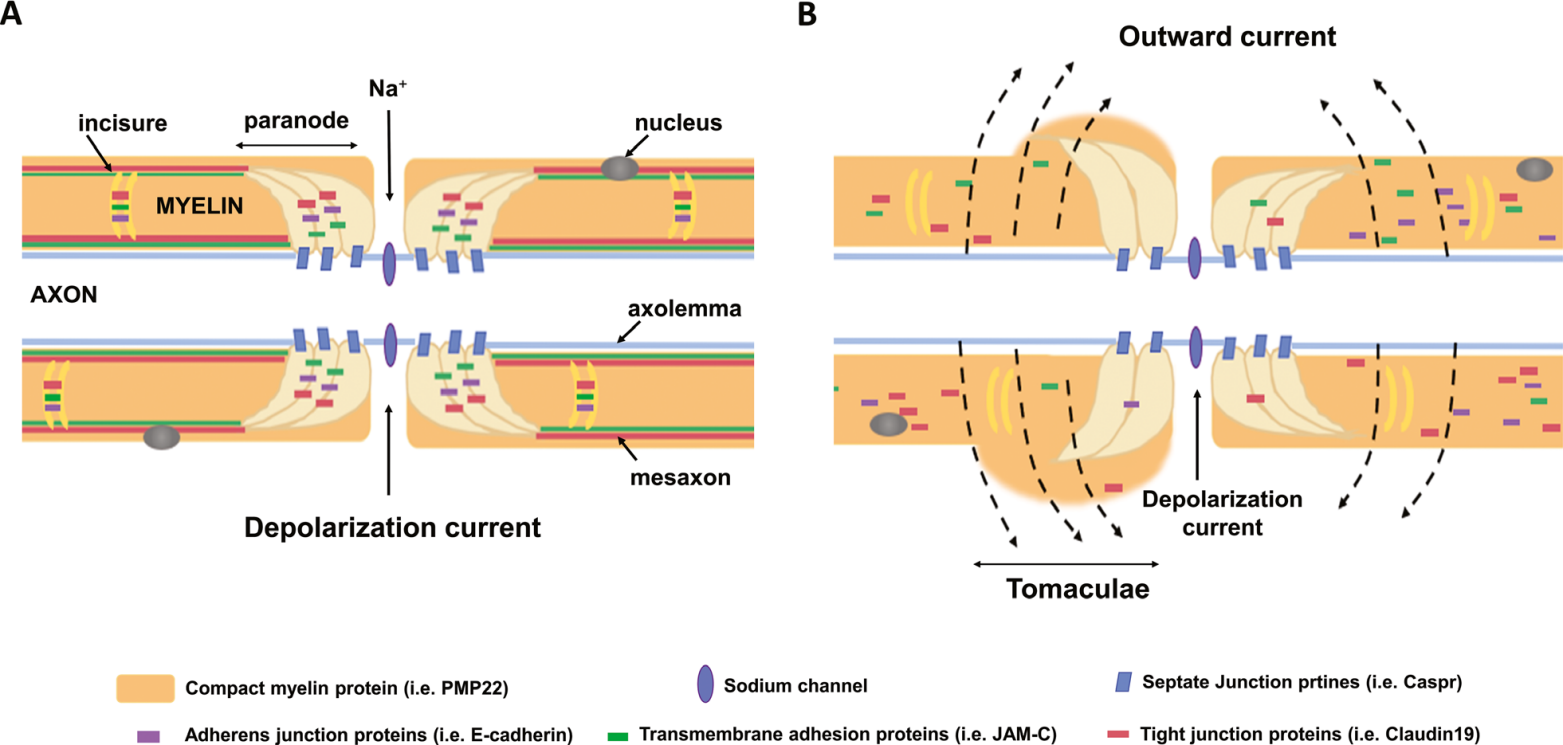
Schematic illustrations of myelin junctions in peripheral nerves. Axonal current in normal (A) and functionally demyelinated nerves in HNPP (B) with increased myelin permeability (modified from Hu et al, PloS Genetics 2016).^6^

Schwann cells in the peripheral nervous system (PNS) wrap axons concentrically in consecutive segments, defining the territory of an individual *internode*. The small gap between internodes forms the node of Ranvier where the voltage gated sodium channels (Na_v_) are concentrated (Figure 4). Schwann cells use *septate junctions* to anchor the paranodal myelin loops onto the axolemma adjacent to the node of Ranvier and thereby define the area of the *paranode*. The small segment (~10µM length) adjacent to the paranode is the *juxtaparanode* and is defined by the distribution of voltage gated potassium channels K_v_1.1 and K_v_1.2.^23^ The remaining internode is covered by compact myelin, which is periodically interrupted by Schmidt‐Lanterman *incisures*. The incisures are non‐compacted myelin tunnels spiraling around the axon.^24^ The incisures are flanked by *tight junctions* and *adherens junctions* that separate them from the compact myelin.^23^, ^25^ Collectively, the paranodal loops and incisures are the *non‐compact myelin*, in contrast to the *compact myelin*.

Myelin seals axons, which enables the fastest conduction of action potentials. This seal also requires myelin junctions to obliterate the spaces between each layers of myelin wrap (Figure 4). Our pre‐clinical studies in HNPP mouse model have demonstrated that haploinsufficiency of *PMP22* in HNPP results in disruption of myelin junctions. The impaired seal of myelin leads to slowed conduction velocity, conduction block, and temporal dispersion in nerve conduction studies. These electrophysiological features recapitulate those seen in acquired demyelinating neuropathies while internodal myelin is still intact in HNPP nerves. We termed this “functional demyelination”.^6^, ^7^ Segmental demyelination (stripping myelin off axon) does not take place until the late stage of the disease in aged HNPP animal model.

In those myelinated nerve fibers with less severe disruption of myelin junctions, conduction block may not occur, but action potential propagation would be at the risk to fail. Intermittent failure of conduction in those nerves would explain fatigue during physical activities. This hypothesis needs to be further tested in the future studies of humans with HNPP.

Our findings also raise several critical issues for the future clinical trials against HNPP: Patients with HNPP exhibit two distinct sets of clinical phenotypes. First, HNPP patients may present with transient, multi‐focal sensory loss or weakness that may or may not be associated with physical activities triggering the focal deficits. Second, axonal loss accumulated over time results in a length‐dependent and symmetric polyneuropathy causing distal limb sensory loss, muscle weakness and atrophy in aged patients. Conventional outcome measurements, such as CMT neuropathy score (CMTNS), were designed based on the length‐dependent and symmetric polyneuropathy seen in other types of CMT. CMTNS would measure the second phenotype; but is not suitable for the first phenotype. Those focal deficits in HNPP may be transient and would introduce unacceptable variations of CMTNS scores, depending on when the scores are collected (at the peak versus at the trough of the episodes). While axonal loss may be measured using techniques like the muscle MRI FF, due to the slow progression of axonal loss in patients with HNPP, sample size may have to be large to detect differences, which is difficult to do due to relatively rare prevalence of HNPP.

Fatigue changes in patients with HNPP appear to be robust in our study. Therefore, fatigue score may serve as an alternative outcome measure if future longitudinal studies can demonstrate good responsiveness, thereby reducing the sample size.

Moreover, in patients with HNPP, fatigue was strongly related to quality of life as measured by the SF‐36 subdomains, including general health, pain, and social functioning. These findings suggest that interventions targeting fatigue have the potential to greatly improve quality of life in patients with HNPP, and are consistent with our prior data in multiple sclerosis showing that fatigue is related to higher pain ratings and poorer quality of life.^26^

Because of the fluctuating nature of HNPP phenotype, the EMA captures day to day variations in fatigue and symptoms over time. EMA fatigue levels are reported five times per day over a 2‐week period on a wrist‐worn device. Unless a severe limb paralysis developed, fatigue score is unlikely affected by transient focal episodes. To ensure no complication from these focal episodes, participants were asked to report limb paralysis with a custom diary during the EMA period, which could be used to rectify large variations (though none were present in this study).

This work is limited by a small sample size (common in rare diseases). Our sample is unintentionally comprised of females only. Because prior work has shown that sexual hormones play a role in regulating PMP22 expression, a single sex of cohort would improve the consistency of the data.

In summary, our study revealed a remarkable level of fatigue in patients with HNPP. This finding is in line with previously reported the impaired safety factor of action potential propagation in PMP22 deficient nerves (functional demyelination).^6^, ^7^ Given the fact that our pre‐clinical study has identified a small molecule compound to treat the functional demyelination,^6^ the present study adds important information how the fatigue score may serve as an outcome measurement in future clinical trials against HNPP. Ongoing efforts by our team are aiming to both recruit a large cohort of HNPP patients for a longitudinal study to further validate these measurements and to elucidate the electrophysiological and histological changes explaining the relationship between fatigue and PD on qMRI in HNPP animal model.

## Conflict of Interest

N.E. Fritz reports no disclosures. Y. Chen reports no disclosures. L. Waters reports no disclosures. S. Saba reports no disclosures. M. Hackett reports no disclosures. F.C. Mada reports no disclosures. J Li is a member in CMT Association Scientific Advisory Committee, an adhoc reviewer in several NIH study sections and provides consultations to Neurogene and Alnylam.

## CRediT Author Statement

**Nora E. Fritz:** Conceptualization, methodology, formal analysis, investigation, writing‐original draft, writing‐ review & editing, supervision. **Yongsheng Chen:** Conceptualization, data curation, methodology, formal analysis, investigation, writing‐ review & editing, visualization. **Lauren Waters:** Data curation, writing‐review & editing. **Sadaf Saba:** Investigation, data curation, writing‐review & editing, visualization. **Melody Hackett:** Investigation, data curation, writing‐ review & editing. **Flicia Christina Mada:** Investigation, data curation, writing‐ review & editing. **Jun Li:** Conceptualization, methodology, formal analysis, investigation, resources, writing‐ review & editing, supervision, project administration, funding acquisition.
